# Enteropathogenic *Escherichia coli* Mediates CoCrMo Particle-Induced Peri-Implant Osteolysis by Increasing Peripheral 5-HT

**DOI:** 10.3389/fcimb.2021.796679

**Published:** 2022-01-05

**Authors:** Kaiwen Xue, Ruijie Tao, Qi Wu, Lei Zhang, Zhongyang Sun, Xing Yu, Jia Meng, Nirong Bao, Jianning Zhao

**Affiliations:** Department of Orthopaedics, Jinling Hospital, School of Medicine, Nanjing University, Nanjing, China

**Keywords:** gut microbiota, aseptic loosening, particle-induced osteolysis, enteropathogenic *E. coli*, 5-HT

## Abstract

The human gut microbiota has been proven to have great effects on the regulation of bone health. However, the association between gut microbiota and particle-induced osteolysis, which is the primary cause of aseptic loosening, is still unknown. In this study, we used a combination of wide-spectrum antibiotics to eliminate the majority of gut microbiota and found that reduction of gut commensal bacteria significantly alleviated the progression of osteolysis, in which anaerobe was the biggest culprit in the exacerbation of osteolysis. Furthermore, colonization of enteropathogenic *Escherichia coli* (EPEC), a subspecies of anaerobe, could promote the development of particle-induced osteolysis by increasing the secretion of peripheral 5-hydroxytryptamine (5-HT) from the colon. Elevated 5-HT level decreased the phosphorylation of CREB and inhibited the proliferation of osteoblasts. Collectively, these results indicated EPEC colonization suppressed the bone formation and aggravated particle-induced osteolysis *in vivo*. Thus, clearance of EPEC is expected to become a potential preventive approach to treat debris-induced osteolysis and aseptic loosening.

## Introduction

Total hip arthroplasty (THA) is considered the most effective surgery performed to treat end-stage joint diseases and severe hip trauma (Goodman et al., 2014; Cherian et al., 2015). Though in some cases imperfect implant design and defective surgical technique can cause arthroplasty failure, periprosthetic osteolysis and secondary aseptic loosening account for the most cause of implant failure and surgical revision (Gallo et al., 2013).

Wear particles, which generate from the abrasion surface of implant components, play a key role in the progression of aseptic loosening (Gallo et al., 2013). It has been extensively studied that wear debris interacts with multiple kinds of cells, like macrophages, osteoclasts, osteoblasts, and so on, around the prostheses and induces chronic inflammatory responses along with periprosthetic osteolysis, which eventually leads to implant aseptic loosening (Lochner et al., 2011; Haleem-Smith et al., 2012; Wang et al., 2015a; Liu et al., 2016). In view of the above mechanism, the current treatment measures for this complication are generally aimed at reducing bone resorption by inhibiting osteoclasts and promoting bone formation by activating osteoblasts, including bisphosphonates, nonsteroidal anti-inflammatory drugs, and TNF inhibitors (Deng et al., 2017a). Unfortunately, these potential approaches could not achieve the desired effect due to their poor clinical performance and various side effects. Thus, there is still a lack of appropriate pharmacology prevention measures for osteolysis and aseptic loosening. Recently, our study found that *Lacticaseibacillus casei*, a common probiotic, could reduce particle-induced osteolysis by mediating macrophage polarization, indicating that the gut microbiota (GM) may be implicated in the progression of aseptic loosening (Wang et al., 2017a).

Human gut microbiota is the largest reservoir of microbial communities in the body (Gordon, 2012). Emerging evidence has shown that the GM is an essential regulator in a large number of intestinal and extraintestinal diseases, including skeletal diseases (Abernathy-Close et al., 2021; Rettedal et al., 2021; Wang et al., 2021). It has been demonstrated that the GM in elderly individuals with low bone mineral density (BMD) differed markedly in the composition and abundance compared with that in healthy controls (Li et al., 2019). What is more, increased BMD and downregulated osteoclast activities along with alleviated inflammatory reaction were detected in germ-free (GF) mice and wide-spectrum antibiotic-treated animals compared with conventionally raised ones, whereas colonization of healthy gut microbiota to GF mice leads to normalized bone characters, indicating the essential role of gut microbiota in bone metabolism (Cho et al., 2012; Sjogren et al., 2012; Yan et al., 2016). However, it is not clear whether the GM dysbiosis can affect the development of aseptic loosening.

Enteropathogenic *Escherichia coli* (EPEC) is the most important *E. coli* pathotype to be implicated in infantile and adult diarrhea through attaching and effacing (A/E) lesion formation on intestinal epithelial cells (Chen and Frankel, 2005). Esmaili et al. (2009) proved that EPEC-associated diarrhea was caused by inhibition of serotonin transporter (SERT) activity, which decreased the maximal velocity of 5-hydroxytryptamine (5-HT, serotonin) uptake. Central 5-HT is a neurotransmitter involved in numerous physiological processes and peripheral 5-HT, which is mostly derived from the intestine, is supposed to be an essential metabolite for the function of gut and a variety of tissues outside of the gut, including the skeletal system (Kode et al., 2012). Previous research showed that GF mice had decreased serum 5-HT level and intestinal tryptophan hydroxylase 1 (TpH1) expression compared with the conventionally raised ones (Wikoff et al., 2009; Sjogren et al., 2012).

In the present study, we first examined the effect of reduction of gut microbiota by multiple wide-spectrum antibiotics used in combination or separately in a particle-induced osteolysis mouse model. Interestingly, both combinative and separate use of antibiotics attenuated CoP-induced osteolysis. Furthermore, through 16s rDNA sequence, we screened a specific anaerobe EPEC and colonized it to antibiotic-treated mice to verify its effect on osteolysis. The results showed colonization of EPEC had a promotive effect on the progression of osteolysis, which was relied on the regulation of peripheral 5-HT level and proliferation of osteoblasts. Our findings suggested a possible mechanism underlying CoP-induced osteolysis and identified clearance of EPEC as a potential therapeutic approach for treating aseptic loosening.

## Materials and Methods

### Reagents

Bovine serum albumin (BSA, A4161), 5-hydroxytryptamine (H9523), and eletriptan hydrobromide (1234453) were purchased from Sigma-Aldrich Co. Ltd. (St. Louis, MO, USA). Fetal bovine serum (FBS, 10099-141) and α-minimum essential medium (α-MEM, 12561056) were obtained from Gibco Co. Ltd. (Waltham, MA, USA). RIPA lysis buffer (P0013B) was purchased from Beyotime Co. Ltd. (Haimen, China). Ampicillin (A100741), vancomycin (A100990), neomycin (A610366), metronidazole (A600633), and amphotericin B (A610030) were purchased from Sangon Biotech Co. Ltd. (Shanghai, China).

### Particle Preparation

CoCrMo particles (CoPs), provided by Dr. Zhenzhong Zhang from the College of Materials Science and Engineering of Nanjing University of Technology, had a mean particle diameter of 51.7 ± 17.44 nm (mean ± standard deviation) detected by transmission electron microscope (TEM). The particles were autoclaved for 15 min at 121°C and 15 psi. Quantitative Limulus Amebocyte Lysate (LAL) Assay (R13025, Charles River, Wilmington, MA, USA) was used to test the quantity of endotoxin with a result of lower than 0.25% EU/ml. The particles were stocked at a concentration of 50 mg/ml in phosphate-buffered saline (PBS; AR0030, Boster Biological Technology Co., Ltd., Pleasanton, CA, USA).

### Bacterial Culture

*Escherichia coli EPEC 026: K60* (CICC 10372), *Escherichia coli K12* (CICC 10003), and *Bacteroides fragilis* (CICC 24309) were purchased from the China Center of Industrial Culture Collection (CICC). EPEC and K12 were cultured under anaerobic conditions in nutrient agar at 37°C. *B. fragilis* was cultured under anaerobic conditions in trypticase soy agar medium at 37°C.

### Particle-Induced Osteolysis Animal Model and Experimental Design

The mice were obtained from the experimental animal center of Jinling Hospital (Nanjing, People’s Republic of China), and all animal experiments conformed with the Chinese legal requirements (the Laboratory Animal Management Regulations [March 1, 2017, the third revision]). Animal experiments were divided into four experiments as follows, group design 1: group I, sham-operated group; group II, CoP-treated group; group III, sham plus antibiotics cocktail-treated (Abx) group; group IV, CoPs plus Abx group; group design 2: group V, CoPs plus control mice fecal transplantation (CtrMT) group; group VI, CoPs plus Abx-treated mice fecal transplantation (AbxMT) group; group design 3: group VII, CoPs plus ampicillin and vancomycin (Amp/Van) group; group VIII, CoPs plus neomycin (Neo) group; group IX, CoPs plus metronidazole (Met) group; group X, CoPs plus amphotericin B (Amp B) group; Group design 4: group XI, CoPs plus EPEC group; group XII, CoPs plus K12 group, group XIII, CoPs plus *B. frigilis* group. Briefly, the mice were anesthetized, and the cranial periosteum was separated from the calvaria by sharp dissection. Fifty microliters (50 mg/ml) of the CoP suspensions were embedded under the periosteum around the middle suture of the calvaria. The animals were then sacrificed, and the calvarial caps were removed by dissecting the bone free from the underlying brain tissue for further analysis.

### Calvaria Culture

Each of the calvaria was arranged in a well of a 12-well plate and cultured with 2 ml Dulbecco’s modified Eagle’s medium (DMEM; Thermo Fisher Scientifc, Waltham, MA, USA) for 24 h at 37°C with 5% CO_2_ as previously reported (Wang et al., 2017b). The culture medium was then collected and stored at −20°C for further testing.

### Micro-CT Scanning and 3-Dimensional Reconstruction Analysis

The mouse calvaria were analyzed with a high-resolution micro-CT (SkyScan1176; SkyScan, Kontich, Belgium). The parameters were set at 18 μm solution and 45 kV and 550 mA of x-ray energy. After reconstruction, we chose a square region of interest around the midline suture for further qualitative analysis. The BV/TV ratio and percentage of total porosity of each sample were measured as described previously (Wang et al., 2015b).

### Sample Collection and DNA Extraction

Fecal samples were collected from all mice for three consecutive days prior to treatment on day 0, on day 7, and prior to surgery on day 14. Fecal pellets weighted approximately 500 mg were collected from each mouse on autoclaved aluminum foil and then were immediately transferred to prelabeled microcentrifuge tubes, flash frozen in liquid nitrogen, and stored at −80°C until further processing.

Frozen stool samples (500 mg) were placed in sterile polypropylene microvials (BioSpec Products, Bartlesville, OK, USA) containing 1 ml InhibitEX buffer and 1 ml of 0.1 mm diameter zirconia/silica beads (BioSpec Products). Samples were homogenized for 2 min using a Mini-BeadBeater. Total nucleic acids were then extracted using the PowerSoil DNA Isolation kit (MoBio, Carlsbad, CA, USA) according to the user manual and stored at −20°C until sequencing.

### 16s rRNA Sequencing and Data Processing

DNA was submitted for sequencing at Shanghai Personal Biotechnology Co., Ltd. (Shanghai, China) at the Next-Generation Sequencing facility using Illumina MiSeq with 2 × 251 bp paired end reads following established HMP protocols. Briefly, universal primers 515F and 806R were used for PCR amplification of the V5–V6 hypervariable region of 16S rRNA gene using a two-step cycling protocol consisting of 50°C for 2 min, 95°C for 10 min, followed by 45 cycles of 95°C for 15 s, 60°C for 1 min. After PCR products were quantified, the completed library was sequenced on an Illumina Miseq platform following the Illumina-recommended procedures.

Data processing was performed using QIIME 1.9.0, with specific processing steps as follows (Bosman et al., 2019).

### Fecal Microbiota Transplantation

Cecal contents were collected from mice treated with nonmedicated water or Abx solution. According to Benakis et al. (2016), with slight modifications, cecal content was resuspended in PBS prepared in autoclaved tap water (2.5 ml per cecum), filtered using a strainer, and stored at −80°C until use. To facilitate colonization of the transplanted flora, recipient mice were pretreated for 1 week with Abx solution prior to administration of 200 μl of cecal extract by oral gavage. Transplanted mice received fecal microbiota gavage administration once two days for 4 weeks before particle-induced osteolysis particle-induced osteolysi (PIO) surgery and 2 weeks after surgery (Benakis et al., 2016).

### Enzyme-Linked Immunosorbent Assay Detection

The 5-HT level was quantified using enzyme-linked immunosorbernt assay (ELISA) kits (Jinyibai Biological Technology Co. Ltd, Nanjing, China). All procedures were performed according to the manufacturer’s instructions.

### Real-Time PCR

Total RNA from calvarial bone was prepared using TRIzol reagent (15596-018, Invitrogen, Waltham, MA, USA) according to the manufacturer’s instructions. As previously described (Wang et al., 2017a), real-time PCR was performed using 2× SYBR Green qPCR Mix (PC01, Zoonbio Biotechnology Co., Nanjing, China) according to the manufacturer’s protocol. Primers for b-actin were used as internal controls. The following primers were used: Tph1, sense: 50-GGACAGGACACACACACACA-30 and antisense: 50-CAAACAGGAGAGCCACTTCA-30; SERT, sense: 50-GGACAGGACACACACA-30 and antisense: 50-CAAACAGGAGAGCCAC-30; cyclin D1, sense: 50-ACAATCCGTGCCACTCACT-30 and antisense:50-TTTCATCGAGAAAGCACAGG-30; and cyclin D2, sense: 50-GAGCTGGTGTAATGGGTCCT-30 and antisense: 50-GAGACCCAGGAAGACCTCTG-30.

### Western Blotting

The cells were lysed in RIPA lysis buffer with a protein inhibitor cocktail for 30 min on ice, and the lysates were centrifuged at 12,000×*g* for 10 min at 4°C. The supernatants were collected, and the protein concentrations were measured using a BCA protein assay kit (PP1002, Biocolor Bioscience and Technology Co., Shanghai, China). Thirty micrograms of each protein were separated by 12% or 15% SDS-PAGE before being transferred to polyvinylidene fluoride membranes (162-0177, Bio-Rad, Hercules, CA, USA). Western blotting was performed using the following primary antibodies: anti-GAPDH (97166, Cell Signaling Technology, Danvers, MA, USA), anti-CREB (9104S, Cell Signaling Technology), anti-phospho-CREB (Ser 133) (9196S, Cell Signaling Technology). The following secondary antibodies were used: horseradish peroxidase (HRP)-conjugated anti-rabbit IgG (Cell Signaling Technology, 7074) and HRP-conjugated antimouse IgG antibodies (sc-2005, Santa Cruz Biotechnology, Dallas, TX, USA). After probing with specific primary antibodies and a HRP-conjugated secondary antibody, the protein bands were detected, and the band density was analyzed using ImageJ 1.41 (National Institutes of Health).

### Cell Viability Assay

Cells were seeded in 96-well plates and were cultured with or without various concentrations of 5-HT before being stimulated with CoPs for 20 h. Subsequently, cell viability was determined using WST-8 staining with a CCK8 (Cell Counting Kit-8) according to the manufacturer’s instructions (CK04, Dojindo, Kumamoto, Japan). Optical density was determined at 450 nm with a plate reader (Thermo Scientific, Multiskan FC, Waltham, MA, USA).

### Statistical Analysis

Results are expressed as means standard error of the means (SEM). Data concerning apoptotic cells analyzed by flow cytometry are expressed as means standard deviation (SD). The differences between groups were analyzed by the Brown-Forsythe test and, if appropriate, by one-way ANOVA followed by Dunnett’s test or Bonferroni test. A *p*-value of less than 0.05 was considered significant.

## Results

### Antibiotic Treatment Ameliorated CoP-Induced Mouse Calvarial Osteolysis

**Figure 1:**
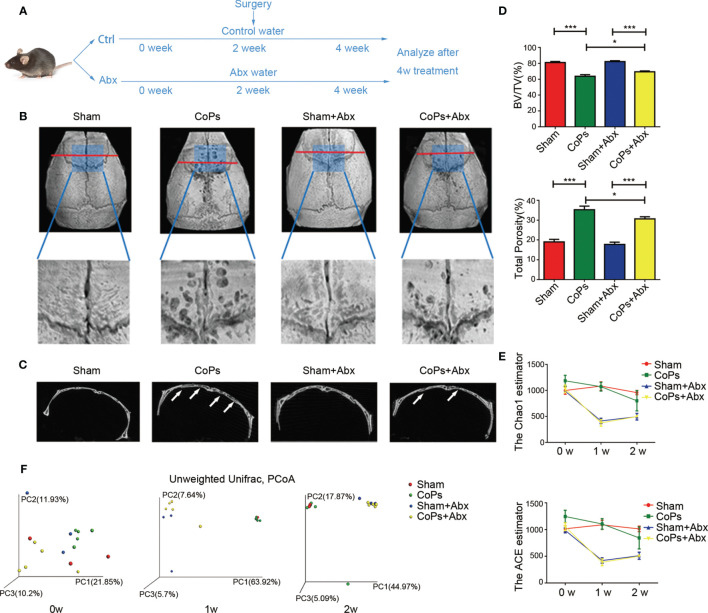
Antibiotics treatment ameliorated CoP-induced mouse calvarial osteolysis. **(A)** The 8-week-old mice were gavaged with antibiotic water or pure water once daily preoperatively for 2 weeks and postoperatively for 2 weeks. After this process, the animals were sacrificed and tissues were collected for further research. **(B)** Representative micro-CT with 3-dimensional reconstructed images and amplified region of interest from each group. The ROI was labeled by a blue square box. **(C)** Representative micro-CT 3D reconstruction cross-section from each group; the arrows marked the area where osteolysis occurred. **(D)** BV/TV and total porosity percentage of each sample were measured. **(E)** The Chao1 and ACE indexes reflecting alpha diversity of four groups examined at points in time of the start, the first week, and the second week. **(F)** PCoA of unweighted UniFrac dissimilarities at three time points with the antibiotic treatment. The data are presented as the mean ± SEM (*n* = 5–7). ^***^ *p* < 0.001.

In order to evaluate the effect of gut microbiota on CoP-induced osteolysis, we used an antibiotic cocktail (Abx), broad-spectrum antibiotics consisting of ampicillin, vancomycin, neomycin, metronidazole, and amphotericin B which were not reported to have an association with osteolysis, to eliminate intestinal microbiota. Abx treatment was administrated by gavage preoperatively for 2 weeks and postoperatively for 2 weeks to clear gut commensal bacteria (**Figure 1A**). The extent of osteolysis was then evaluated by microcomputerized tomography (micro-CT). As shown in **Figures 1B, C**, representative three-dimensional reconstruction (3D reconstruction) images indicated Abx administration mitigated CoP-induced osteolysis. Quantitative analysis of bone parameters further confirmed that Abx treatment increased the bone volume/total volume (BV/TV) index and decreased the porosity percentage (**Figure 1D**).

In order to test the antibacterial efficacy of Abx, fecal microbiota of each group at three specific timing (0 week, 1 week, 2 weeks) was sequenced to analyze the composition and abundance of the microbial communities. The results showed that the Chao1 and ACE index (predictors of species richness based on rare species number) significantly decreased in alpha-diversity after Abx treatment for 1 week (**Figure 1E**). To examine the variability of microbial communities between groups, we calculated the PCoA of unweighted UniFrac dissimilarities (a qualitative indicator of phylogenetic measures of community beta diversity). The results confirmed significant differences between Abx and non-Abx treatment groups after the first week (**Figure 1F**). These findings suggested that modulating gut commensal bacteria can specifically ameliorate calvarial osteolysis induced by wear particles.

### Transplantation of Fecal Microbiota From Abx-Treated Mice Attenuated CoP-Induced Osteolysis

**Figure 2:**
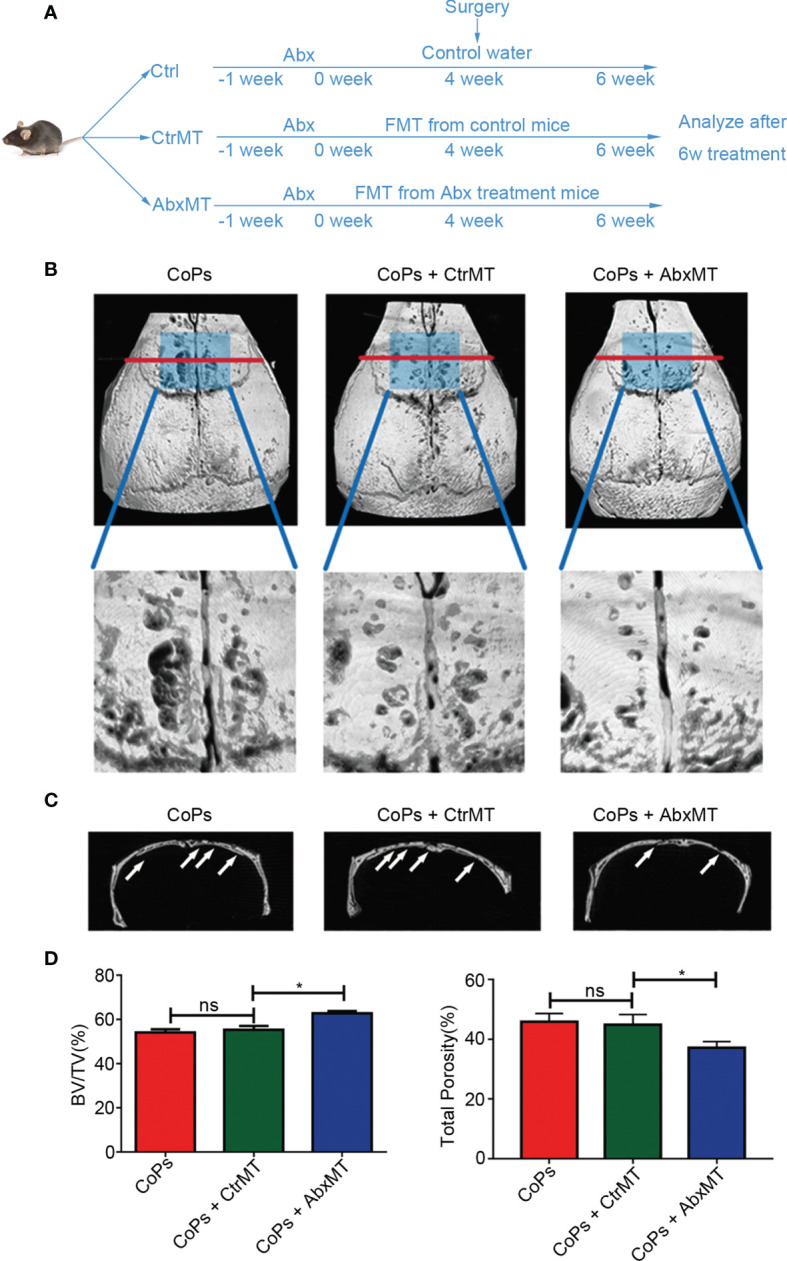
Transplantation of fecal microbiota from Abx-treated mice attenuated CoP-induced osteolysis. **(A)** The 6-week-old mice, which were pretreated with Abx for 1 week, were transplanted with fecal microbiota from Abx-treated mice (AbxMT) or normal mice (CtrMT) once every 2 days preoperatively for 4 weeks and postoperatively for 2 weeks. The mice were then sacrificed for later analysis. **(B)** Representative micro-CT with 3D-reconstructed images and amplified ROI from each group. The ROI was labeled by a blue square box. **(C)** Representative micro-CT 3D reconstruction cross-section from each group; osteolysis areas were marked by the arrows. **(D)** BV/TV and total porosity percentage of each sample were measured. The data are presented as the mean ± SEM (*n* = 5–7). ^*^ *p* < 0.05; ns, *p* > 0.05.

Previous researches suggested antibiotic treatment could inhibit osteoclastogenesis and periprosthetic inflammation *in vivo* (Ren et al., 2009; Liu et al., 2014). To rule out the influence of Abx directly modulating bone remodeling of osteolysis, we transplanted fecal microbiota collected from conventionally raised and Abx-treated mice to the particle-induced osteolysis mouse model prestimulated by Abx for 1 week. Experimental design is illustrated in **Figure 2A**. The osteolysis result assessed by micro-CT and 3D reconstruction images showed that mice in Abx-treated transplantation group had milder extent of osteolysis compared with that in normal-raised transplantation group (**Figures 2B, C** **)**. Consistent with the previous results, quantitative analysis of bone parameters confirmed microbiota transplantation from Abx-treated mice significantly increased the BV/TV ratio and decreased the total porosity percentage (**Figure 2D**). The data further indicated that the attenuation of calvarial osteolysis induced by CoPs was a benefit from gut microbiota modulation rather than the use of Abx.

### Colonization of EPEC Aggravated CoP-Induced Mouse Calvarial Osteolysis

**Figure 3:**
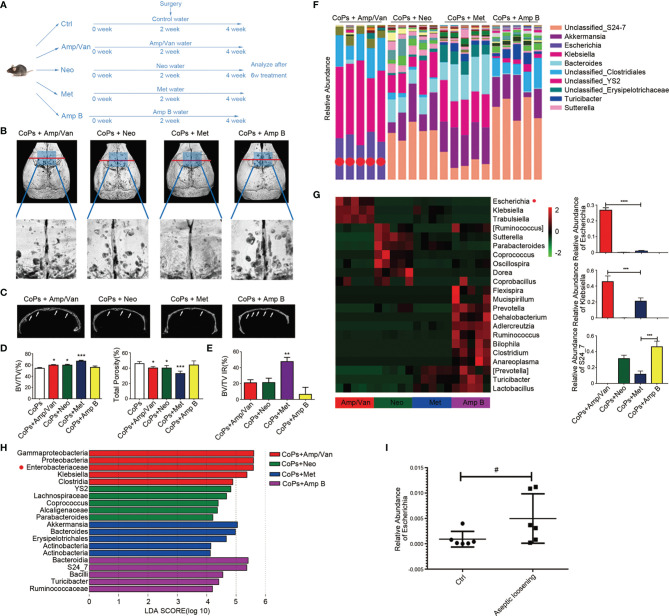
Anaerobic bacteria contributed most to the progression of CoP-induced osteolysis. **(A)** The 8-week-old mice were given four different kinds of water containing antibiotics by gavage to eliminate different bacteria once per day preoperatively for 2 weeks and postoperatively for 2 weeks. The animals were then sacrificed for further analysis. **(B)** Representative micro-CT with 3D-reconstructed images and amplified ROI from each group. The ROI was labeled by a blue square box. **(C)** Representative micro-CT 3D reconstruction cross-section from each group; osteolysis areas were marked by the arrows. **(D)** BV/TV and total porosity percentage of each sample were measured. **(E)** The improvement rate (IR) of BV/TV of each group was calculated. **(F)** Taxonomic composition and community abundance distribution map of genus level was drawn by R software. The abundance bar column of *Escherichia* was marked by red dots. **(G)** Heat map showed the top 22 bacteria community abundance at genus level in four groups. The relative abundance of *Escherichia*, *Klebsiella*, and S24_7 were calculated. **(H)** LEfSe analysis showed key bacteria community in each group. The threshold for the logarithmic LDA score was 4.0. **(I)** The relative abundance of *Escherichia* of control patients (Ctrl) and aseptic loosening patients examined by metagenomic sequencing were calculated. The data are presented as the mean ± SEM (*n* = 5–7). ^*^ *p* < 0.05; ^**^ *p* < 0.01; ^***^ *p* < 0.001; ^****^ *p* < 0.0001; ^#^ *p* = 0.0649.

Since gut microbiota was involved in the progression of osteolysis, we tried to figure out the specific bacteria strains in the GM that had an effect on particle-induced osteolysis. According to group design 3 in **Table 1**, five antibiotics in the cocktail were divided into four treatment groups according to their individual antimicrobial spectrum (Amp/Van for gram-positive bacterium, Neo for gram-negative bacterium, Met for anaerobe, and Amp B for fungus) (**Figure 3A**). Representative 3D reconstruction results of micro-CT illustrated that mice in Amp/Van and Neo-treated groups had significantly higher BV/TV index and lower total porosity than that in the CoP-treated group, while Amp B treatment did not affect the calvarial osteolysis (**Figures 3B–D**). The most increased BV/TV ratio was in the Met-treated group, and the extent of osteolysis was distinctly raised by 23.81% ± 2.658%. (**Figure 3E**), which indicated anaerobic community eliminated by metronidazole was the biggest culprit behind the progression of osteolysis.

**Table 1:** Experimental design.

Group	Antibiotic treatment	CoPs	Microbiota transplantation
**Group design 1**			
Sham			
CoPs		Yes	
Sham+Abx	Abx		
CoPs+Abx	Abx	Yes	
**Group design 2**			
CoPs	Abx pretreatment	Yes	
CoPs+CtrMT	Abx pretreatment	Yes	Microbiota from control mice
CoPs+AbxMT	Abx pretreatment	Yes	Microbiota from Abx-treated mice
**Group design 3**			
CoPs+Amp/Van	Amp/Van	Yes	
CoPs+Neo	Neo	Yes	
CoPs+Met	Met	Yes	
CoPs+Amp B	Amp B	Yes	
**Group design 4**			
CoPs	Abx pretreatment	Yes	
CoPs+EPEC	Abx pretreatment	Yes	EPEC
CoPs+K12	Abx pretreatment	Yes	K12
CoPs+*B. fragilis*	Abx pretreatment	Yes	*B.fragilis*

Study groups design for Experiments 1, 2, 3, and 4.

We then sequenced the V5–V6 hypervariable region of 16s rRNA genes from each antibiotic-treated group for the taxonomic research. The taxonomic distribution of four groups in the genus level showed the dominant bacteria communities in each group (**Figure 3F**). Also, the top 22 upregulated bacteria genus in other three groups than that in Met-treated group was listed in the heat map (**Figure 3G**). We detected 20 key microbial communities with the criteria of LDA score (Linear discriminant analysis Effect Size (LEFSe)) >4 (**Figure 3H**). Furthermore, we found that *Enterobacteriaceae* was the most significantly altered genus, which was consistent with the metagenomic sequencing results from microbiota of aseptic loosening patients (**Figure 3I**).

**Figure 4:**
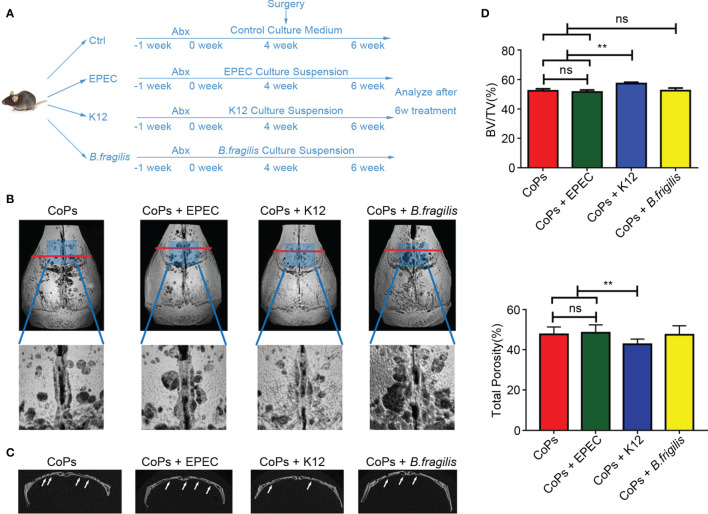
Colonization of EPEC aggravated CoP-induced mouse calvarial osteolysis. **(A)** The 6-week-old mice, which were pretreated with Abx for 1 week, were administrated with culture medium containing EPEC, K12, or *B. fragilis* by gavage once every other day preoperatively for 4 weeks and postoperatively for 2 weeks. The animals were then sacrificed for further test. **(B)** Representative micro-CT with 3D-reconstructed images and amplified ROI from each group. The ROI was labeled by a blue square box. **(C)** Representative micro-CT 3D reconstruction cross-section from each group; osteolysis areas were marked by the arrows. **(D)** BV/TV and total porosity percentage of each sample were measured. The data are presented as the mean ± SEM (*n* = 5–7). ^**^ *p* < 0.01; ns, *p* > 0.05.

Based on the above taxonomic results, we chose to transplant mice with *Escherichia coli* K12 (K12, a representative apathogenic subspecies in *Enterobacteriaceae*) and enteropathogenic *Escherichia coli* (EPEC, a representative apathogenic subspecies in *Enterobacteriaceae*) to explore their effects in particle-induced osteolysis. Meanwhile, we also selected *Bacteroides fragilis* (*B. fragilis*, a representative species in *Bacteroides*), which makes up the most substantial portion of anaerobic. The experiment procedure is illustrated in **Figure 4A**. Representative 3D reconstruction images of micro-CT revealed that the osteolysis induced by CoPs was aggravated by colonization of EPEC compared with that of K12 (**Figures 4B, C** **)**. However, colonization of *B. fragilis* had no significant difference with that of EPEC (**Figures 4B, C** **)**. Quantitative analysis also indicated reduced calvarial BV/TV index and rising total porosity percentage in the EPEC-colonized group compared with the K12-colonized group (**Figures 4D, E** **)**. Additionally, EPEC colonization did not cause statistical difference of weight loss compared with conventional raise. Interestingly, there was no difference in the extent of osteolysis between mice in the control group and that in the EPEC-colonized group, which meant EPEC colonization alone could simulate the effects of CoP-induced osteolysis as whole gut microbiota did.

### EPEC Increased the Level of 5-HT by Inhibiting the Expression of Sert *In Vivo*

**Figure 5:**
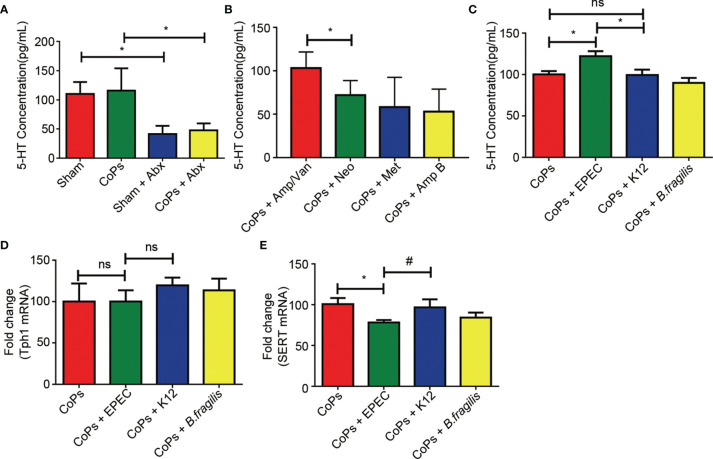
EPEC increased the level of 5-HT by inhibiting the expression of SERT *in vivo*. Notes: The level of 5-HT when treated with antibiotics **(A, B)** and colonized with EPEC **(C)** was examined by ELISA assay. The expression of Tph1 **(D)** and Sert **(E)** mRNAs from each group was examined using real-time PCR. The data are presented as the mean ± SEM (*n* = 5–7). ^*^ *p* < 0.05; ns, *p* > 0.05; ^#^ *p* = 0.13.

Since it has been reported that gut microbiota could mediate the serum level of 5-HT by regulating the activation of Tph1 and SERT, we then examined the level of 5-HT in calvaria from mice of the above experimental groups. As shown in **Figures 5A, B**, both combined and separate use of EPEC-sensitive antibiotics markedly downregulated the level of 5-HT. When EPEC was directly colonized, the 5-HT concentration has remarkably risen compared with that of the CoPs and K12-colonized groups (**Figure 5C**). No significant difference was detected between the CoPs group and the K12-colonized group (**Figure 5C**). As 5-HT secretion is generally regulated by TpH1 and SERT, we examined their expression in the colon. The results showed that the expression of TpH1 remained unchanged while the level of SERT was significantly downregulated after administration of EPEC compared with that of CoPs and K12 (**Figures 5D, E**). Collectively, the promotive effect of EPEC on peripheral 5-HT secretion was through inhibiting SERT expression *in vivo*.

### 5-HT Inhibited the Proliferation of Osteoblasts Through pCREB/Cyclin D *In Vivo* and *In Vitro*

**Figure 6:**
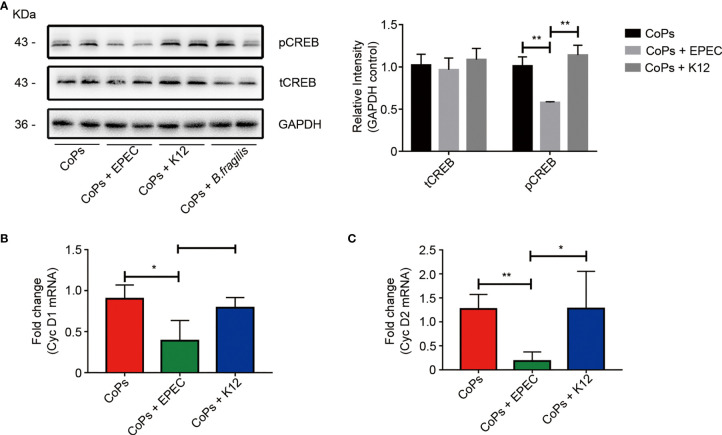
5-HT inhibited the proliferation of osteoblasts through pCREB/Cyclin D *in vivo*. **(A)** Western blots performed after calvaria from mice colonized with EPEC, K12, and *B. fragilis* were collected. The density of the Western blot bands was quantified using Image J software. The level of Cyc D1 **(B)** and Cyc D2 **(C)** mRNAs from each group were examined using real-time PCR. The data are presented as the mean ± SEM (*n* = 5–7). ^*^ *p* < 0.05; ^**^ *p* < 0.01.

As gut-derived 5-HT is capable of inhibiting osteoblast proliferation by downregulating the phosphorylation of CREB, we then investigated the expression of CREB and phosphorylated CREB in calvaria of mice in different groups. No obvious difference of total CREB expression but a significant decrease of the phosphorylated CREB expression was detected in EPEC-colonized group compared with that in CoPs and K12-colonized groups (**Figure 6A**). Previous studies indicated that phosphorylated CREB specifically regulated the expression of cyclin proteins (Chen et al., 2020), we next investigated whether EPEC colonization affected the cyclin D expression. The RT-PCR results suggested that both levels of cyclin D1 and cyclin D2 in calvaria were downregulated in EPEC-colonized group compared with that in CoPs and K12-colonized groups (**Figures 6B, C** **)**.

**Figure 7:**
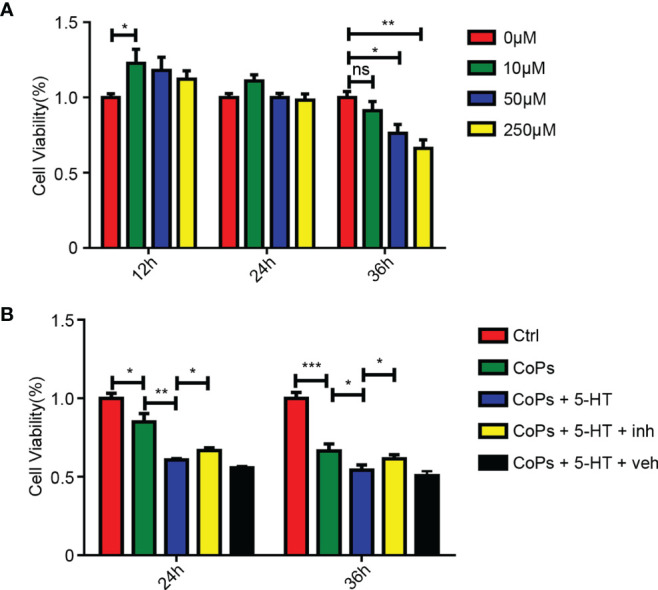
5-HT mediated the proliferation of osteoblasts through Htr1b receptor *in vitro*. **(A)** Cell viability of MC3T3-E1 after treated by different concentrations of 5-HT at three different time periods was calculated by CCK8 method. **(B)** After cotreated with CoCrMo particles, 5-HT, and 5-HT receptor inhibitor (inh), the cell viability of MC3T3-E1 was calculated by CCK8 method. ^*^ *p* < 0.05; ^**^ *p* < 0.01; ***p < 0.001; ns, p > 0.05.

To further confirm the effect of 5-HT in the proliferation of osteoblasts, we used 5-HT to stimulate osteoblast MC3T3-E1 cells. The cell viability of osteoblasts treated with different concentration of 5-HT was examined at different time points. CCK8 assay showed that 5-HT inhibited the proliferation of osteoblasts in a dose- and time-dependent manner (**Figure 7A**). We then used eletriptan hydrobromide, a selective 5-HT1B receptor agonist, to costimulate osteoblasts with 5-HT. According to **Figure 7B**, the downregulation effect of 5-HT on the proliferation by CoP stimulation was markedly attenuated after treatment with eletriptan (**Figure 7B**). These results further confirmed that the proliferation of osteoblasts was suppressed by treatment of 5-HT.

## Discussion

Total hip arthroplasty is widely acknowledged as the most useful surgical procedure to treat various end-stage joint diseases. However, the ratio of revision after hip arthroplasty still remains at a high level with aseptic loosening accounting for the largest part of surgical failure. Wear particles are regarded as a critical role in the pathogenesis of aseptic loosening and osteolysis by interacting with multiple cell types from the peri-implant tissue and resulting in various biological reactions (Wang et al., 2013; Wang et al., 2015a; Wang et al., 2017a). Most studies on particle-induced osteolysis have focused on the role of local factors in mediating bone resorption and bone formation (Deng et al., 2017b; Liu et al., 2020). While our present study suggests that gut microbiota, a distant organ of the skeletal system, stimulate the progression of osteolysis in mouse model and EPEC take an important part in the promotion of aseptic loosening.

Specifically, after administration of Abx in particle-induced osteolysis model (**Figures 1A–D**), we found Abx treatment effectively eliminated the majority of gut microbiota and alleviated CoP-induced osteolysis in the mouse model. However, Liu et al. (2014) also used oral administration of enoxacin to treat particle-induced osteolysis mouse model and found potent inhibitory effect on periprosthetic osteolysis *via* suppression of osteoclastogenesis. In addition, Ren et al. (2009) suggested that erythromycin treatment improved the inflammation status and inhibited the cytokine release in peri-implant tissue of aseptic loosening patients. They believe that antibiotics directly mediate the formation of osteoclast and inhibit inflammation in local tissue. To eliminate the direct effect of Abx on bone metabolism, only the fecal microbiota of gnotobiote and conventionally raised mice were transplanted to experimental groups. Our results suggest that the regulation of Abx on the progression of osteolysis is through the gut microbiota rather than influence of osteoclasts (**Figures 2A–D**). Sjogren et al. (2012) reported that germ-free mice had higher BMD and decreased osteoclastogenesis activities compared with the conventionally raised mice. What is more, colonization of GF mice with normal gut microbiome normalized bone mass (Sjogren et al., 2012). Schwarzer et al. (2016) suggested the bone formation was also significantly downregulated in germ-free mice. These conclusions were also demonstrated by the study of Uchida et al. (2018). Their results showed germ-free mice had alleviated both osteoclast and osteoblast activities by regulating specific transcription factors. Interestingly, they suggested that the commensal microbiota prevented excessive mineralization by mediating osteocalcin expression in osteoblasts (Uchida et al., 2018). In an antibiotic-treated gnotobiote mouse model, Yan et al. (2016) suggested that deleting gut flora inhibited bone formation by decreasing serum insulin-like growth factor 1 (IGF-1). On the other side, not only gut microbiota can regulate particle-induced osteolysis, but particle challenge can affect the balance of gut microbiota (Moran et al., 2020). Collectively, we demonstrate that gut microbiota mediate CoP-induced osteolysis, but the effect of CoP challenge in our experiment on the GM remains to be further explored.

Through the separate use of Abx, we narrowed the target scope to several specific bacterial kinds, *Enterobacteriaceae*, *Klebsiella*, and S24-7 (**Figures 3F–H**). After comparing with the metagenomic sequencing results of clinical aseptic loosening patients, we finally picked out a potent genus, *Escherichia*. We found colonization of EPEC in mice remarkably aggravated the extent of osteolysis (**Figures 4A–D**). Previous research found that EPEC isolates can be identified as typical (tEPEC) or atypical (aEPEC) according to the existence of eaeA and bfpA genes. Also, most diarrheal stool samples of adults were infected by aEPEC, part of which were asymptomatic infection (Carlino et al., 2020). Furthermore, it was suggested that among immunosuppressed cancer patients, EPEC was easier to infect and carried higher burden of EPEC with antibiotic-resistant strains (Olvera et al., 2021). These results indicate although EPEC is not easy to infect adults, it may still play a role in aseptic loosening patients, especially those with immunosuppression or intestinal microbiota dysbiosis.

The underlying mechanism of osteolysis and aseptic loosening was proved through increasing the secretion of peripheral 5-HT and downregulating the proliferation of osteoblasts (**Figures 5** and **6**) The bone-bowel connection was firstly elucidated by Yadav et al. (2008), and they reported that gut-derived 5-HT inhibited bone formation by downregulating osteoblast proliferation. 5-HT is generally regarded as a neurotransmitter in the central nervous system (CNS) involved in activities such as emotional regulation. However, the vast majority of 5-HT is located in the gut and regulates the gut motility, secretion, and vasodilation function, which is known as conventional function of 5-HT. Meanwhile, peripheric 5-HT can play important roles in other tissues out of gut, including hematopoiesis, metabolic homeostasis, and bone metabolism, namely, nonconventional function of 5-HT (Spohn and Mawe, 2017). Kousteni et al. further found evidence for the gut-bone axis by demonstrating that FOXO1 inhibited osteoblast proliferation *via* regulating the expression of 5-HT (Kode et al., 2012). In this study, EPEC augmented the level of 5-HT in the mouse calvarial tissue and promoted CoP-induced osteolysis by restraining CREB phosphorylation and osteoblast proliferation *in vivo* and *in vitro*.

Nowadays, the metabolic potential of gut microbes and their effects in the regulation of human health is emerging. Advancement in microbiology has inspired the use of additive, subtractive, and modulatory therapies of microbiome engineering in clinics, in which probiotic treatment is the most important part (Cullen et al., 2020; Yadav and Chauhan, 2021). Probiotics, which are live commensal microorganisms providing health benefits and improving the gut flora, are claimed to be useful on the protection of bone loss (Uchida et al., 2018). Fukuda et al. (2011) suggested that *Bifidobacterium* can protect from EPEC infection. In addition, our previous study suggested that *L. casei* could reduce particle-induced osteolysis by decreasing the M1-like macrophage/M2-like macrophage ratio (Wang et al., 2017a). Collectively, administration of probiotic, especially EPEC-fighting strains, in aseptic loosening patients may also be a potent treatment approach.

There are some limitations of this study. First, we only selected a specific strain among the whole bacteria community with higher abundance than that in the metronidazole-treated group, and we think there are much more bacteria worthy of study among them. Secondly, we hope to further verify the effect of gut flora on particle-induced osteolysis by transplanting the intestinal flora of aseptic loosening patients and normal patients. However, based on the results of our previous metagenomic sequencing of gut microbiota from aseptic loosening patients, the human intestinal flora varies greatly with region, age, and diet. In further research, enough fecal samples of aseptic loosening patients and their direct relatives with the same habits need to be collected to eliminate experimental disturbance.

## Data Availability Statement

The data presented in the study are deposited in the NCBI Sequence Read Archive database, the both accession numbers are PRJNA775674 and PRJNA775886.

## Ethics Statement

The studies involving human participants were reviewed and approved by the Ethics Committee of Jinling Hospital. The patients/participants provided their written informed consent to participate in this study. The animal study was reviewed and approved by the experimental animal center of Jinling Hospital.

## Author Contributions

KX, JZ, and NB conceived and designed the study. KX, RT, and QW performed experiments and analyzed the data. ZS and XY wrote and edited the manuscript. LZ and JM provided materials and advice on data interpretation. All authors contributed to the article and approved the submitted version.

## Funding

This work was supported by the National Natural Science Foundation of China (8157211).

## Conflict of Interest

The authors declare that the research was conducted in the absence of any commercial or financial relationships that could be construed as a potential conflict of interest.

## Publisher’s Note

All claims expressed in this article are solely those of the authors and do not necessarily represent those of their affiliated organizations, or those of the publisher, the editors and the reviewers. Any product that may be evaluated in this article, or claim that may be made by its manufacturer, is not guaranteed or endorsed by the publisher.
